# Exploring the paradoxical nature of cold temperature mortality in Europe

**DOI:** 10.1038/s41598-024-53675-z

**Published:** 2024-02-07

**Authors:** Marcin Piotr Walkowiak, Dariusz Walkowiak, Jarosław Walkowiak

**Affiliations:** 1 Department of Preventive Medicine, Poznan University of Medical Sciences, Święcickiego 6, 60-781 Poznań, Poland; 2 Department of Organization and Management in Health Care, Poznan University of Medical Sciences, Poznań, Poland; 3 Department of Pediatric Gastroenterology and Metabolic Diseases, Poznan University of Medical Sciences, Poznań, Poland

**Keywords:** Environmental impact, Natural hazards

## Abstract

While low winter temperatures are associated with increased mortality, this phenomenon has been suggested to be most severe in regions with seemingly mild winters. The study aimed to establish a temperature-based formula that could elucidate the previously ambiguous regional differences in vulnerability to low temperature. European weekly mortality data (2000–2019) were matched with meteorological data to determine for each region vulnerability to temperature decrease and the optimal temperature with lowest mortality. Regression models were developed to generalize and explain these findings considering regional temperature characteristics. Optimal temperature could be predicted based on local average summer temperature (R^2^ = 85.6%). Regional vulnerability to temperature decrease could be explained by combination of winter and summer temperatures (R^2^ = 86.1%). Regions with warm winters and cold summers showed the highest vulnerability to decrease of temperature during winter. Contrary to theories about economic disparities Eastern Europe exhibited resistance comparable to Scandinavia. The southern edges of Europe demonstrated serious low temperature vulnerability to decreased temperatures, even if temperature was relatively high around 20 °C. This suggests that the observed connection primarily reflects the modulation of the length of respiratory virus infection seasons by climate conditions, counterbalanced by varying levels of acquired immunity and the presence of heatwaves eliminating the most frail individuals. Thus, relatively low vulnerability and a flat mortality cycle in countries with harsh climates paradoxically imply the presence of threats throughout the whole year.

## Introduction

Observed differences in vulnerability to temperature are often attributed to local cultural or infrastructural adaptations to the prevailing conditions^1^. This interpretation finds some support in prior studies, which have noted regional variations in the optimal temperature for humans, with warmer regions generally exhibiting slightly higher optimal temperatures^2,3^. However, attempts to identify the actual factors that contribute to significant disparities have proven challenging. The Eurowinter Group conducted a comparative study involving European populations and found very faint differences. For instance, at a temperature of 7 °C, Finnish houses maintained a slightly higher average temperature than Greek (21.7 °C vs. 19.2 °C), and although the clothing surface area was similar, Finns were more likely to wear hats or gloves^4^. While this explanation implies acclimatization to a usual temperature range, it is noteworthy that the primary contributor to temperature related mortality is not extreme cold or heat, but rather moderate cold temperatures^3^. Furthermore, the theory that local populations are optimally adapted to their climate struggles to account for cases when the annual cycle is interrupted, as years with exceptionally mild winters correspond to lower mortality rates^5,6^. Conversely, in the aftermath of lifting COVID-19 restrictions, those same countries experienced out-of-season epidemics that were no longer suppressed^7^. Additionally, local adaptations alone cannot explain the clear pattern of late summer and early autumn having the lowest mortality, while the same conditions are not as favorable a few months earlier, which is especially noticeable in warmer countries with higher annual mortality amplitudes^8,9^.

While the increasing number of infections during winter is uncontroversial, the interpretation of their relationship with cold-related mortality remains contentious within the literature. Certain studies openly acknowledged influenza epidemics, but consider it as confounding factor when analyzing impact of cold weather on mortality^4,10^. Conversely, other findings highlighted influenza or other infections as a significant contributing factor to cold-related mortality^3,11^. Moreover, some research either openly suggest that additional mortality in winter is primarily caused by infections^12^ or implicitly, by successfully estimating the death toll associated with common viruses^13^. The mortality estimates attributed to nine well-monitored viruses are significant enough to account for a substantial portion of the excess mortality observed during winter. Furthermore, the superposition of these viruses' impact exhibits a resemblance to the overall observed mortality cycle^13^.

There are multiple studies suggesting a potential relationship between winter mortality and social deprivation, which is clearly correlated on regional level. While such a regional relationship was observed in Portugal, it lacked age control^14^. Considering the economic migration of young people, it is expected that the remaining population of retirees would exhibit a higher unadjusted mortality rate due to the age composition. Moreover, when crudely controlling for climate and age structure in a US dataset, the relationship does not appear to be robust, as various causes of death are predicted by distinct socio-economic markers^15^. Additionally, initial theories proposing fuel subsidies as a factor in reducing winter mortality in Britain^16^ have been challenged, with subsequent research suggesting that the observed difference is primarily attributable to variations in influenza strains^12^. There is a study indicating a correlation with a mix of climatic, socio-economic, and infrastructure factors, which, on the surface, seems methodologically sound. However, this study leaves unanswered questions, particularly regarding its model's suggestion that winter mortality falls with double glazing but rises with floor isolation^17^. Furthermore, in the case of analyzing the north–south divide, there are intertwined observations of climate factors, climate-adapted infrastructure and economic disparities.

Previous works measured regional differences in annual mortality cycles but were less successful in generalizing the underlying mechanism. They notably detected a relationship between Excess Winter Mortality (EWM) and climate^18^, with the highest mortality amplitude observed around latitude 35, gradually decreasing towards the equator and poles^12,19^. Additionally, while analyzing the roles of climate and economic factors, prior works did not leverage the natural experiment provided by Eastern Europe, which combines the harsh climate of Northern Europe with the economic development of Southern Europe. The purpose of this article is to investigate whether the observed regional differences in optimal temperature and vulnerability to low temperatures could be attributed to simple climatic patterns. The detection of such patterns could provide valuable insights into the underlying mechanisms driving these variations.

## Methods

### Data source

For this study, mortality data in ISO-weeks for NUTS1 regions from Eurostat for the period 2000-W01 to 2019-W52, covering 72.9 million deaths, were selected^20^. These data were combined with information from the European Climate Assessment and Dataset^21^, which contains data from 7050 meteorological stations. The regional climate data were based on the period from 1995-W01 to 2019-W52.

### Calculation

Weather stations located above 1250 m were excluded due to their lack of representativeness for major population centers. To account for potential projection and rounding errors encountered with stations located on the edges of regions (such as sea shores or minor mountain peaks), a tolerance level of 10 km was applied during the matching process. This allowed them to be linked with more than one region if applicable, resulting in the creation of a dataset comprising 7130 region/station pairs. Subsequently, the regional time series underwent a deduplication process. For each region, the daily median temperature across stations was calculated to mitigate the impact of extreme observations, and from these medians, a weekly average temperature was derived.

While previous studies often focused on average annual^14^ or winter temperatures^4,15,16^, we took inspiration from the Köppen–Geiger climate classification system^22^. This system typically employs two proxy variables related to the average temperatures of colder and warmer seasons. For modeling consistency, we had to standardize these seasons length owing to their diverse durations in the original classification. To ensure robustness and accommodate minor regional variations in the timing of warmer and colder seasons, we calculated average temperatures by considering observations above or below the regional median, further referred to as summer and winter temperatures.

Previous studies have indicated that the specific temperature measure used does not appear to be relevant, but properly considering the lag is highly relevant^23^. However, the lagged impact of high and low temperature is different. Heatwaves cause death almost immediately; however, they are followed by around 2 weeks of below-trend mortality, as there is observed a very strong harvesting effect^24,25^. Especially during less intense heatwaves, the majority of casualties are expected to include individuals in the final days of their lives, and it would be desirable not to overestimate this impact. The impact of low temperatures is mediated through an indirect, delayed mechanism. The relationship between mortality and temperature displays a peak around a single-week lag^1,11^ before gradually diminishing, with noticeable impact diminishing after 3 weeks^1,5^. To further complicate the issue some works suggest that the best fit lag undergoes some subtle fluctuations over annual cycles^26,27^. The lag of 3 weeks, typically used in the literature^1–3,5,28^, seems to be the most suitable approach, though due to mortality data being available in weeks, this means comparing mortality in week n against temperature in weeks n to n-2.

**Figure 1:**
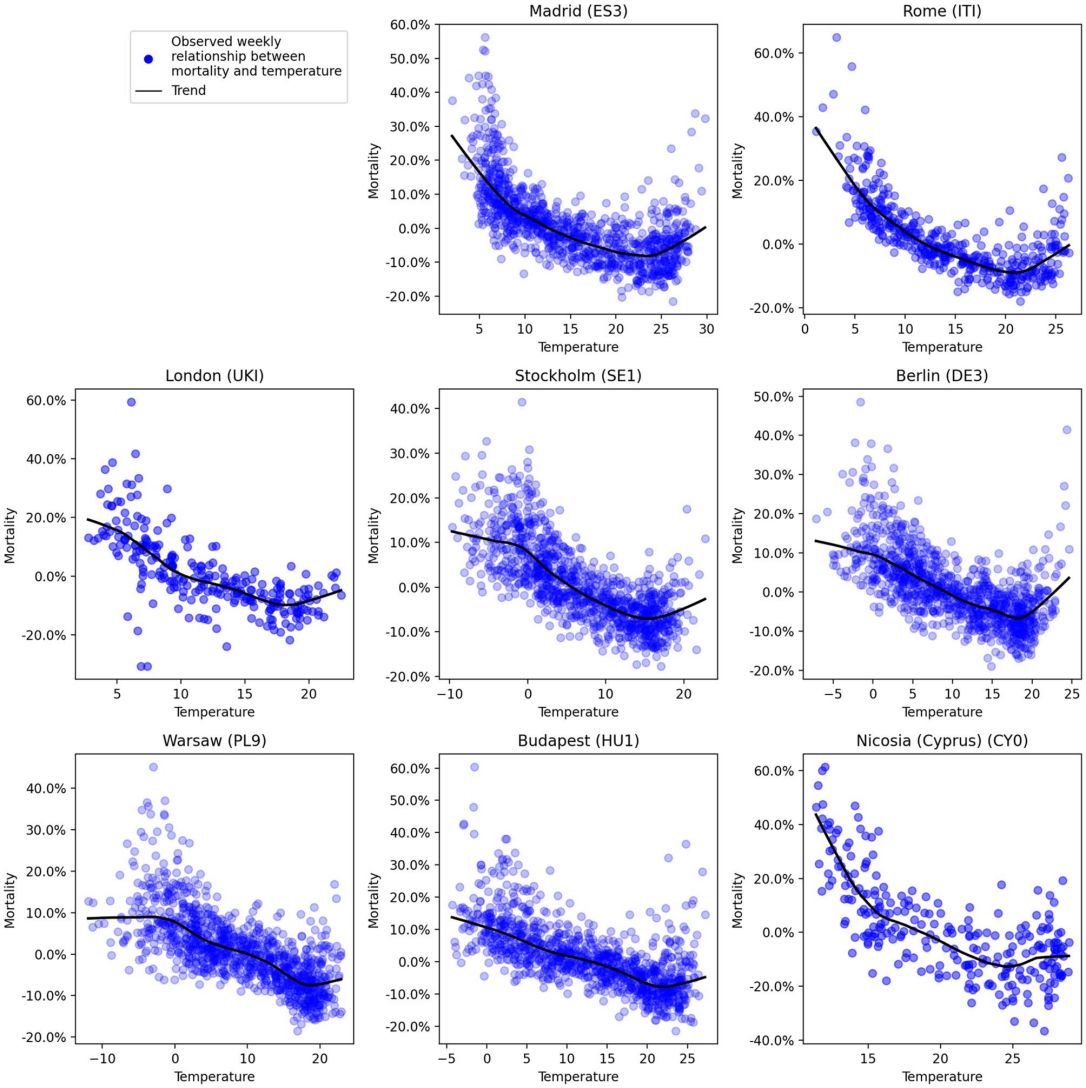
Relationship between weekly temperature and percentage of mortality deviation from the trend line in selected European regions.

**Figure 2:**
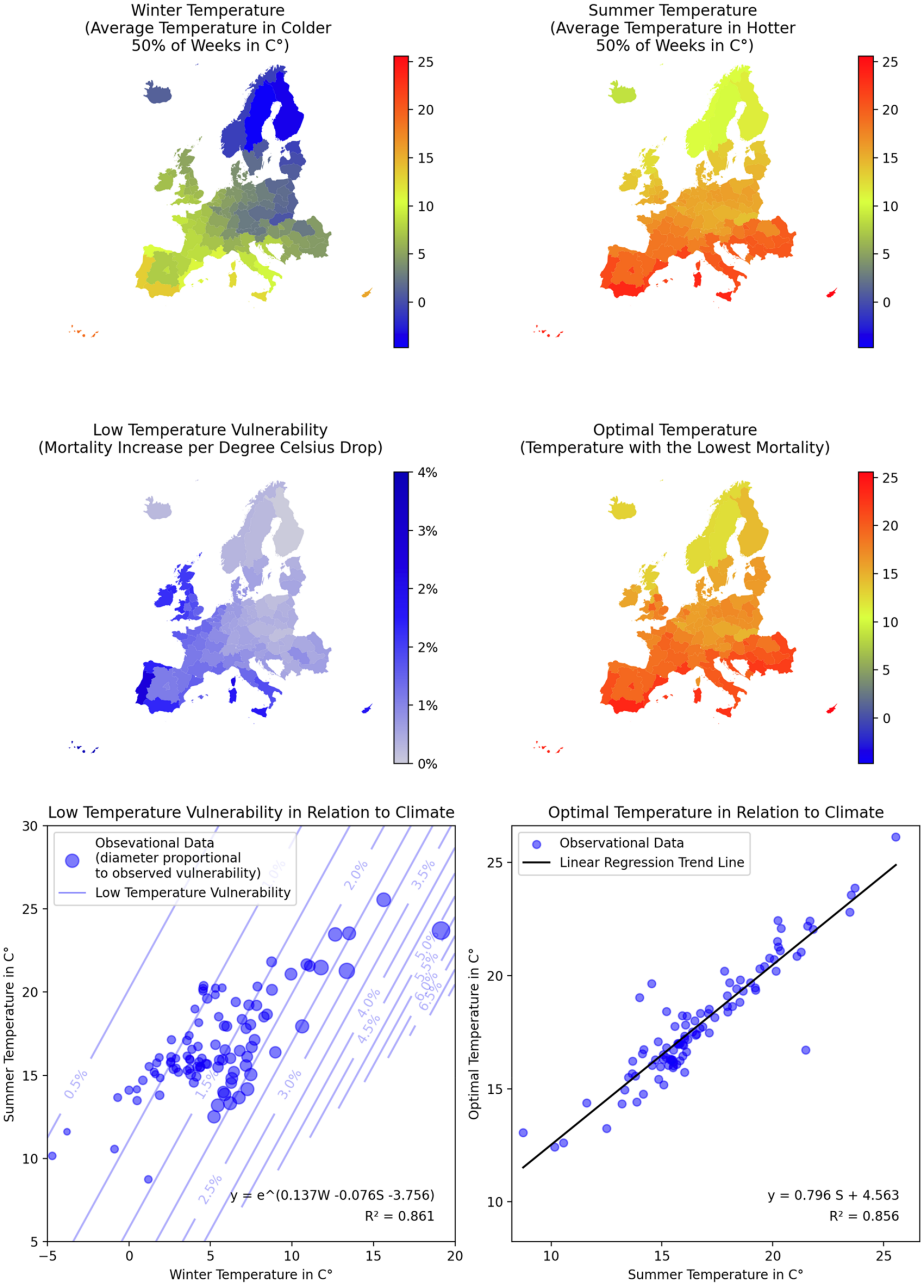
Relationship between climate and low temperature vulnerability in Europe.

Logarithmic normalization was applied to regional weekly mortality data and they were decomposed using STL (Seasonal and Trend decomposition using Loess) to eliminate regional trend and magnitude. This allowed for the extraction of the relative seasonal component and relative residuals. The resulting data was then transformed into percentage deviation from the trend. These regional time series were plotted alongside the corresponding temperature data for each region, as depicted in Fig. 1 for selected regions. The selection of the presented areas was based on those that aligned with the study conducted by Gasparrini et al.^3^, with the inclusion of a few additional from eastern part of the European continent. Loess trend lines were employed to depict the relation between mortality. The optimal temperature for each region was identified as the temperature corresponding to the lowest mortality rate, after excluding observations falling within the first and last percentile. A subset of observations within the temperature range from 1 °C to the optimal temperature was selected for each region to calculate the slope using the Theil–Sen estimator. Subsequently regression models explaining optimal temperature and low temperature vulnerability based on climate variables were calculated, what is presented in Fig. 2.

The calculations were performed in Python 3.10 with its inbuilt library math and using libraries such as pandas 1.4.3 and numpy 1.21.1. The map was processed using geopandas 0.13.2, and the coordinates were converted using pyproj 3.5.0. The weather stations were matched with regions using shapely.geometry 1.7.1. Trends were calculated using statsmodels 1.13.5 and numpy, and the results were visualized using matplotlib 3.7.1 and geopandas. 95% confidence intervals (CI) were employed.

## Results

The association between the average temperature of the preceding three weeks and mortality changes compared to the baseline was depicted in Fig. 1. Employing Loess instead of splines maintained the characteristic U-shaped trend, albeit with a less pronounced slope due to reduced sensitivity to extreme values. Notably, at both the lower and upper ends of the temperature range, mortality exhibited not only an overall increase but also a growing upward dispersion, leading to the selection of the central band for further analysis. At low temperatures, increased dispersion was attributable to the modulation of winter infections without affecting the severity of circulating strains. Conversely, high temperatures exhibited a harvesting effect, predominantly impacting individuals who were already frail, resulting in somewhat lower mortality in subsequent weeks in spite of continuously elevated temperature. Contrary to economic-based theories, cities like Warsaw or Budapest exhibited robust resistance to low temperatures, similar to Stockholm or Berlin. Within the typical temperature range, there was a nearly linear trend between decreasing temperature and increasing mortality. Remarkably, the deadly impact of low temperatures was evident in the southern edges of Europe even around 20 °C.

Figure 2 illustrates the climate conditions of European regions and their correlation with vulnerability to low temperatures and the temperature at which mortality is the lowest. The temperature gradient in Europe is influenced not only by the north–south difference in solar irradiance but also by a discernible east–west divide due to the Gulf Stream. This warm oceanic current stream provides the western part of the continent with a milder climate, characterized by unusually warm winters relative to their latitude. This geographical pattern aligns well with the vulnerability to low temperatures, explaining why the mortality pattern observed in London resembles that of Madrid more than Stockholm. The vulnerability to low temperature followed an exponential relationship with climate factors. High winter temperature was the main predictor (coefficient: 0.137; CI 0.124–0.148), while high summer temperature was observed to reduce vulnerability (coefficient: − 0.076; CI − 0.092 to − 0.061). Additionally, there was a constant component (coefficient: − 3.756; CI − 3.967 to − 3.545). Given the exponential nature of the relationship, regions with warm winters and relatively cold summers experience heightened vulnerability to cold. Furthermore, the optimal temperature is 79.6% (CI 73.0% to 86.2%) of the average summer temperature in Celsius plus 4.56 °C (CI 3.44 to 5.68).

## Discussion

The observed phenomena can be aptly explained by considering basic climate variables. While the traditional focus of inquiry regarding winter mortality revolves around the puzzling question of why seemingly almost favorable conditions can turn staggeringly deadly in certain regions, a shift in perspective reveals a more suitable question. It becomes important to understand the conditions that allow individuals to live long enough to become vulnerable. The model provides an answer by suggesting that people fortunate enough to reside in climates where they neither face harsh winters nor heatwaves are particularly likely to have a higher chance of surviving until they become frail and succumb in less than optimal conditions. Conversely, in harsher climates, the lower annual amplitude of mortality also becomes easily explainable, as the conditions are unfavorable for most of the year. Similarly, at temperatures decreasing below zero the linear relationship starts to break as if the factor has already exhausted its impact.

The majority of our findings are in line with previous studies. Results for Eastern Europe were highly consistent with those of Germany or Scandinavia, indicating that the observed patterns in these countries followed climate rather than economic development level. However, the use of Loess instead of splines in our analysis inadvertently leads to somewhat different conclusions regarding the impact of high temperatures. In previous studies, a very steep increase in mortality was observed due to high temperatures. In our study, we find that the impact of temperatures deviating above the optimum was comparable to the impact of temperatures deviating below the optimum. In case of more robust fitting, provided a more balanced representation of the data as prolonged periods of warm spell tended to have mortality below the baseline. We consider the previous fitting predominantly as a visualization of the outlier bias, as prior models effectively predict an impending and deadly natural disaster if such weather conditions persist. However, in countries where these conditions do persist, they instead result in an increase in the optimal temperature. Even with temperatures reaching levels considered high by local standards, there is only moderately higher number of fatalities observed.

As the lag for low temperature and deaths necessitates an intermediate factor, respiratory viral infections exhibit many parallels. For instance, the lag between a decrease in temperature and an upswing in respiratory visits is strikingly similar^29,30^. While these viruses typically peak during winter, this seasonal pattern is most pronounced in intermediate climate zones but not in northern latitudes^31^ or equatorial^32^. The onset of the recent COVID-19 pandemic served as a natural experiment to elucidate the modulation mechanism by providing interruption of a non-climatic nature, followed by an ultimate significant rebound in infections. During the initial phase of the pandemic, due to restrictions and voluntary behavior changes, respiratory infection visits^33,34^ and mortality reduced^7,35^. This was accompanied by a decreasing detectable antibody level for common viruses^36^. Nevertheless, in the aftermath of the lifting of pandemic restrictions, there was an extraordinary and out-of-season rebound of those infections^37,38^, with the increase in mortality being the strongest in countries that were the most successful in containing the pandemic^7^.

While only a minority of EWM deaths are officially certified as caused by respiratory infection, there is a well-known issue of underdetection, even with the best-monitored viruses, which becomes pronounced during pandemic waves. While the COVID-19 pandemic highlighted underdetection^39–41^, in influenza pandemics, only a mere one fourth of influenza-associated deaths were even officially associated with respiratory illness^42,43^. Additionally, the same causes of death that were highly likely to be selected for influenza-associated deaths are the ones that show annual fluctuation, peaking throughout winter^4,18,44^.

Analysing the observed vulnerability to low temperature as epidemiological phenomenon starts to explain observed paradoxes. Within this framework prior works trying to establish whether the key to containing numerous concurrent respiratory outbreaks number lies in improving housing conditions^14^, fuel subsidies^16^, installing double glazing^17^ or donning slightly warmer clothing^4^ might seem a bit unconventional. Instead, the primary determinant for any endemic virus would be the underlying level of acquired immunity modulated by weather conditions^29,45^. This mechanism would be able to explain staggering differences in vulnerability between regions with shorter or longer infection season.

The model highlights an additional mechanism unrelated to infection spread, revealing that warm winters reduce vulnerability to cold, challenging the concept of adapting to a specific temperature range. A similar pattern of the most frail people finding some stressors partially interchangeable is well-documented: seasons with more deadly influenza correlate with subsequent reductions in heatwave deaths^25,46–48^, or notably deadly heatwaves lead to detectable lower mortality the following winter^49^. Despite earlier studies recognizing a broad connection between climate, particularly winter temperature, and EWM, the significance of summer temperature through the harvesting effect of heatwaves seems to have been neglected.

While prior studies have suggested a broad optimal temperature around the 75th percentile^1^, indicating some general trends, regional studies have revealed a more nuanced climate relationship^50^. This variation spans from the 60th percentile in tropical areas to approximately the 80–90th percentile in temperate regions^3^. This is consistent with the estimation for our sample at the 81st percentile, with a standard deviation of 5.9 percentiles. To the best of our knowledge, our contribution lies in being the first to formulate a predictive model for this nuanced climate relationship, shedding new light on the understanding of optimal temperature patterns.

Our findings significantly impact the interpretation of the impact of global warming, especially in light of Gasparrini et al.'s predictions, which suggest a significant increase in heat-related deaths due to global warming, combined with a modest reduction in winter deaths^2^. While they acknowledge assuming no adaptation, in light of our findings, they effectively assume that over the span of a century, the local climate-based relationship would remain constant despite the changing climate. Moreover, for the observed local relationships to hold under changing climate conditions, requires additionally existence of an unobserved mechanism in regions that currently experience infrequent heatwaves, ensuring the replenishment of a pool of highly vulnerable individuals every week. However, studies on the impact of heatwaves have found a completely opposite phenomenon. Heatwaves tend to lead to a harvesting effect, where mortality falls below the trend line in subsequent weeks, as those who were the most vulnerable have already succumbed^24,25^.

The assumption of a lack of adaptation is also hard to reconcile with literature as demonstrable cases of population suffering elevated mortality due to lack of adaptation, required to analyze migrants who moved to countries 10 °C colder than their country of origin^51^. Lifting the assumption that the local climate-based relationship would remain constant over the long run would paint a somewhat different picture of our future. After a challenging-to-quantify adjustment period, the new local relationship would be simply based on the new climate as derived from the formula. A warmer summer should lead to higher locally optimal temperatures, resulting in only a slight rise in the number of high-temperature deaths. Conversely, a warmer winter should shorten the infection season but make it more intense, paradoxically leading to an elevated number of deaths in winter in relation to the annual minimum. While this suggestion may seem paradoxical, that's exactly what we observe right now in the Mediterranean climate^8,19^.

Even with this enhanced extrapolation, it is most likely to not include the most important caveat. While the overall pattern of annual mortality cycles seems to have largely stabilized in the last half-century, suggesting a period of relative consistency, earlier dynamics clearly hinted that moderately high temperatures used to be a major environmental hazard before the widespread adoption of modern sanitary standards^9,52,53^. Nevertheless, even in recent decades, our resilience to environmental hazards is steadily increasing, as there has been both an observed decrease in EWM^35,54^ and a reduction in vulnerability to heatwaves^54–56^. Thus, the future relationship is likely in flux and depends not only on our climate but also on the relative success in mitigating specific threats.

## Conclusion

The observed regional differences in optimal temperature and vulnerability to low temperature are clearly shaped by the local climate and are unlikely to remain constant under climate change. The linear relationship between summer temperature and optimal temperature implies that spline-based extrapolation is highly likely to overestimate mortality from temperature increase. As the relationship is typically climate-based, it was disrupted by COVID-19 restrictions. This strongly suggests that the observed connection primarily reflects the modulation of respiratory virus infection cycles by climate conditions. Differences in the length of the infection season, varying levels of acquired immunity, and the existence of other stressors eliminating the most frail individuals would well explain the underlying process. This would suggest that the situation is not most severe in countries with high EWM but paradoxically in those countries with harsher climates, as a flatter cycle and low vulnerability imply the steady presence of threats over the whole year.

### Limitations

The model exclusively focused on calculating the impact of temperature, while characterizing climate under the Köppen–Geiger classification system includes three variables, with the omitted one being precipitation^22^. Although precipitation is likely to play a role, it is more challenging to model, given the unique preferences of particular viral strains. For example, influenza A is known to favor low humidity, while influenza B exhibits a preference for high humidity^57^. While we consider the most plausible interpretation of the role of warm summers as heatwave deaths, our model effectively detected climate continentality. Thus, it would be possible that there were some other coexisting, less conspicuous climate-related risks that, for model purposes, were indistinguishable, like higher daily temperature amplitudes. Attempting to incorporate these relationships would introduce additional layers of complexity with limited prospects for significantly enhancing predictive accuracy. For some countries, time series were relatively short (e.g., 5 years for the UK). Although it did not affect the fitting, as bootstrapping did not cause any noticeable differences due to the extreme robustness of Theil–Sen estimator, there is an underlying issue of representativeness.

## Data Availability

The datasets analyzed during the current study are available in the Eurostat repository (mortality) https://ec.europa.eu/eurostat/databrowser/product/view/demo_r_mwk2_ts and ECAD (mean temperature) https://knmi-ecad-assets-prd.s3.amazonaws.com/download/ECA_blend_tg.zip
